# Allegheny County Medical Society

**Published:** 1889-04

**Authors:** 


### SOCIETY REPORTS.

### *ALLEGHENY COUNTY MEDICAL SOCIETY.*

*Special Meeting, February 19th, 1889.*

W. F. Knox, M.D., President, in the Chair.

Dr. Lincoln reported a case of

FEVER FOLLOWING CONFINEMENT.

Upon the ninth day, after a tedious but otherwise normal, instrumental delivery, resulting in a slight perineal laceration, I was called to see the patient, a slight woman, and found her with a temperature of 103.8°, and back and hip ache. Pain and temperature elevation disappeared during the following three days, but both with a pulse of 140 returned on the sixth day. The fever now assumed an irregularly intermittent type, and now, 20 days from its beginning, convalescence is present. The labor was conducted with antiseptic precautions. Her treatment was intra-uterine bichloride injections, quinine and morphine for pain and sleep.

Dr. Samuel Ayres read a paper on

PARACENTESIS IN INTERNAL HYDROCEPHALUS.

The history of the boy, who is present this evening, is as follows: His age is nearly five years, his height is 3 feet 1½ inches, and weight 43 pounds. There seems to be no history of the neuroses in his family. His father's mother died of cancer of the breast, and an aunt of his mother of phthisis. His mother and father have always enjoyed the best of health, the latter being a laborer. He denies any venereal infection.

The mother states that her boy was perfectly healthy and normally developed up to three months of age, when, without known cause, he was suddenly seized with convulsions during sleep. These attacks became very frequent, occasionally from twenty to thirty occurring daily, and they continued for nine months, or until he was one year old. They then ceased, and have not recurred. Three months after their commencement his head had naturally enlarged and assumed a pear shape, the greater diameters corresponding with the biparietal parieto-occipital planes. There was no separation of the cranial bones. From the date of the beginning of his trouble up to the time that he was brought to my clinic, in October, 1888, every form of treatment had been faithfully tried, according to his parents, but without the least success. At that time his condition was this: Mentally, there had been little or no development. He was obviously imbecile. He could not talk, but smiled idiotically. He was totally blind, but the other special senses were not apparently affected. He had never walked or stood alone, but could easily move his body and extremities. The bowel and bladder sphincters were not controlled. He was extremely irritable and restless, and slept very irregularly. He would take only liquid food given from a spoon. He was fairly developed physically and quite well nourished, but always of an ashy pallor. There was a very frequent rotary movement of the head, with slight retraction, and grinding of the teeth. His pulse was from 120 to 140 per minute. Temperature was not taken. The measurements of the head were the following: From the gabella to inion, 12½ inches; over the biauricular line, 13¾ inches; around the fronto-occipital line, 20 inches. The anterior fontanelle closed when he was eighteen months old, and the sutures had ossified at the usual time. No abnormal sensory symptoms were noticed. We could not succeed in making an examination of the eyes owing to his restless state.

A diagnosis of ventricular effusion was ventured. His parents suggested and urged some kind of an operation as a *dernier ressort*, as they fancied something might be done surgically. To this I very reluctantly consented, having distinctly pointed out the dangers and uncertainties incident to such procedure.

On the 4th of December, 1888, assisted by Drs. Murdoch, Hersman and Boreland, and in the presence of the students of the Western Pennsylvania Medical College, the following course was pursued: The head having been shaved, washed with strong soap, and enveloped in carbolized cotton for twenty-four hours, the boy was chloroformed. Over the coronal suture, about one and one-half inches to the right of the median line, a small flap of the scalp and pericranium was reflected. With a trephine about one cm. in diameter, I removed a button of bone, which was slightly thicker than normal. The dura looked healthy, but owing to its small exposure the tension could not well be estimated. It distinctly pulsated. A very delicate trocar was passed through the dural membrane into the brain substance, downwards, backwards and inwards, to the depth of two and one-half inches, the object being to pierce the central cavity of the right lateral ventricle.

There were no reflex movements of any kind observed during this procedure. The trocar being removed, a clear, limpid fluid began to ooze, drop by drop, from the canula. About an ounce of this was evacuated, when the canula was slowly withdrawn. As its internal orifice reached the sub-dural space, the same transparent fluid dropped from the opposite end, thus revealing the presence of perhaps an excess of fluid in this space also.

Its specific gravity was not obtained, but it was faintly alkaline, non-albuminous, contained chlorides in abundance, and also phosphates. Evidently it closely resembled the cerebro-spinal fluid.

The pericranium and scalp were approximated and stitched, and the wound dressed with carbolized gauze. For several days the same fluid continued to ooze from the puncture in the dura, and to saturate the dressings. It was estimated that from four to eight ounces was thus discharged.

The case progressed satisfactorily, the pulse not exceeding 140, nor the temperature rising above $101\frac{8}{10}°$. The child seemed anxious to be up in two or three days, and could then stand alone. The results of the operation were quickly observed in a partial restoration of sight. The boy could evidently see, as he would wink when a finger was passed before his eyes. Then, having stood unassisted, he was gradually able to walk alone across the room, which he did in about three weeks. He became less irritable, and instead of short snatches of sleep, as before, slept the entire night. He also took some solid food, which he had not done previously. For three weeks following the tapping he did not rotate the head a single time.

The mother states that, in a great many respects, the child is improved. He is more attentive, and seems to better understand her. There is, however, no development of speech, nor are the sphincters under any better control.

He continued in this improved state till the latter part of January last, when, after some slight ailment, he gradually lost the power to walk. Thinking that the fluid was reaccumulating, we proposed tapping him again on the 8th of February, but on the 5th he commenced to walk again, so it was postponed.

It is my opinion that more fluid will have to be evacuated, as he is not quite so active now as some time after the tapping. I am fortunate in being able to report an ophthalmoscopic examination of his eyes, which was very kindly made to-day by Dr. Lippincott, while the boy was anæsthetized for the purpose.

"Right eye. Disc snow white, and with sharply defined margins. Capillary circulation greatly diminished. Retinal vessels, especially veins, reduced in calibre. Arteries not very much below normal size. Retina also atropic. Media clear.

"Left eye. Same condition as right, except that the disc margins are even more sharply defined and a small quantity of pigment is seen surrounding the disc.

"Divergent strabismus of O. S. Pupils normally react."

The most practical bearing this operation would seem to have is in reference to those imbecile children, the commencement of whose trouble dates back to some acute or subacute cerebral affection, as simple meningitis or convulsions, or perhaps slight traumatism, but whose cases have always been considered irremediable. It seems probable that many such cases go to fill the institutions for feeble-minded throughout our land, or else prove, in their own homes, a grave care and an eternal responsibility to parents. And the question may be pertinently asked whether an excessive or a pathological effusion within the cerebral ventricles or sub-dural space, or within both, may not only have caused, but perpetuated, these undeveloped brains and minds.

The presence of such excess of fluid is abundantly adequate to produce the symptoms by pressure upon the delicate nerve elements or tracts, and the varying character of the attacks, as in this case, would strengthen the probability that a fluid, changing with the body position or blood-pressure, was the responsible agent in their production. Or, during the sub-acute stage, when convulsions, coma, paralysis, or other grave symptoms follow an obscure brain attack, consisting, perhaps, in ventricular inflammation, and when death is imminent, it seems to me not only justifiable but imperative, that the sub-dural space be tapped, and if the symptoms do not soon abate, that the lateral ventricles be penetrated. If the product of such inflammation should prove to be purulent, which is not probable, the opening in either case would have to be enlarged and thorough drainage, with antiseptic irrigation, established.

But since most of these sub-acute cases of sub-dural or ventricular meningitis are attended only with fluid effusion, the operation would be comparatively easy, particularly if the fontanelles had not ossified. I shall never again stand by, even after exhausting all other resources, and allow convulsions or coma or paralyzed respiratory centers to carry off an infant, with all the symptoms of ventricular effusion, without resorting to paracentesis, unless a tuberculous history makes certain its fate.

In the performance of the operation great care should be exercised. The chief difficulty lies in our inability to determine which cavity to evacuate. For instance, if the fluid resides in both cavities, and the normal openings between them, through the foramen of Majendie, and those behind the roots of the glosso-pharyngeal nerves be closed by inflammatory exudation, or the presence of a tumor, then to tap only the sub-dural space would remove the external pressure, and allow such an expansion of the internal fluid as would perhaps lacerate the brain tissue. Or the same effect might be produced by evacuating only the ventricular fluid. This may have been the cause of death in some of the reported cases.

It would seem, therefore, that the safest course to pursue would be that followed in the above, particularly when the cranial sutures are closed--to tap first the ventricles and then the external space.

The operation of tapping for hydrocephalus is not new. It is thought Le Cat first performed it in 1744. West (3d *Am. Ed.*, p. 117) refers to the operation, but states that opinion is divided as to the propriety of the practice. He had up to that time collected 56 reported cases from different sources, with 4 recoveries, but he does not state whether they are of the external or internal form; though the inference is that they were of the former, since compression after operation is referred to.

Watson (p. 291 *et seq.*) gives several cases of the external form, for which tapping was practiced, with variable results, some dying, some recovering, but he speaks of no cures.

Ellis (3d Ed., p. 83) says that puncturing is not applicable to the ventricular form, and he rather discourages its employment in the external. He quotes Churchill, who gives a list of unsuccessful cases. But Ellis admits that the operation has been occasionally successful.

In the Cyclopædia of Practical Medicine (vol. II, p. 500) Folis is quoted as giving the names of 27 writers who had expressed themselves in favor of the operation. Yet he himself with Boerhaave, Dupuytren, Heister, Hecker and Portenschlag, regarded it as cruel and useless.

George B. Wood (2d Ed., vol. II, p. 353) says that tapping has been employed by many with uncertain results.

Elliotson (2d Ed., p. 511) states that he never saw a case of the kind, but that if a minute puncture be made and a small quantity of fluid be evacuated at a time, it might be done with safety and with prospects of relief.

Reynolds (*System of Med.*, vol. I, p. 840) quotes Watson, West and Conquest on the subject, the last of whom he states has been the greatest advocate of the operation in this country (England).

Condie (2d Ed., p. 400) refers directly to the tapping of the ventricles and points out with remarkable accuracy the procedure. At the same time he admits that he had never seen a case.

Trousseau (*Clinical Med.*, vol. I, p. 890) remarks that the brain has been tapped through the sutures and fontanelles for hydrocephalic accumulation, but he does not seem to think its advantages counterbalance its disadvantages.

Henoch (*Handbook for Phys. & Student*, p. 116, Wm. Wood & Co., 1882) says of chronic internal hydrocephalus: "At least I have achieved no results whatever, either with mercurial inunction, iodide of potassium or applications of tincture of iodine to the head. Nor can I promise any results from compression of the skull with strips of adhesive plaster, or *from puncture through the fontanelles.*"

Gross (5th Ed., vol. II, p. 123) has practiced paracentesis in two cases, both dying within two days after the operation.

Agnew (vol. II, p. 886) speaks of the benefits of the operation as claimed by West and Conquest, but declares such results have never been obtained in this country. He describes only the tapping of external hydrocephalus when the sutures or fontanelles are not closed.

Meigs and Pepper (7th Ed., 1882, p. 557) refer to West's cases and give one of internal hydrocephalus, upon which they operated, death following in less than forty-eight hours. Examination, however, proved that the case was hopeless, the brain being disorganized at the base, and the child, less than three years old, the victim of miliary tuberculosis.

J. Lewis Smith (6th Ed., pp. 448 and 449) advises the performance of the operation, and regards it as simple and devoid of danger, but his remarks apply only to the congenital form, and evidently to those with opened sutures and fontanelles. He makes no reference to tapping in acquired hydrocephalus.

Gowers (*A Manual of Diseases of the Nervous System*, vol. II, 1888, p. 544) considers evacuation by puncture the most direct, but unfortunately the most dangerous, treatment, and advises that but a small quantity be let out each time, and compression of the skull by elastic bandages be kept up. This procedure, he says, is of course most suitable in external effusion, but it has been employed in the ventricular also, occasionally without ill effect, but with absolute success only in rare instances.

An operation in a different kind of case altogether, but with very similar technique, excepting that in this the dura was incised, is referred to by Senn,* an imperfect account of which is obtained from the *Mel-*

* Annual of the Universal Medical Sciences, Vol. II, page 36.

*bourne Age*, a non-professional Australian journal.

This operation was done for an echinococcus cyst in the brain on January 27, 1888. The patient, a girl of sixteen, was chloroformed. A button of bone one inch in diameter was removed from the left temple, the dura incised, and a trocar inserted through the substance of the brain, and the cyst successfully penetrated, a large quantity of fluid coming away.

Thus it will be seen that a diversity of opinion is expressed by authors as to the propriety of paracentesis capitis. But in the light of modern antisepsis, and of a more intimate knowledge of the brain anatomically and physiologically, it would seem that this operation must take its legitimate place among those which were once conceived to be both impracticable and impossible.

I can find reference to no other case similar to the one above reported, in which tapping was practiced for both ventricular and sub-dural effusion of the primary or acquired character, the sutures and fontanelles being closed, and trephining being required to permit of the entrance of the trocar.

Dr. Murdoch: I saw Dr. Ayres' patient, and assisted him at the operation. I saw the child but once—at that time. He is certainly improved physically; from my slight acquaintance with his pecularities, I venture the opinion that he is much more animated and gives greater evidence of physical skill.

Dr. Thomas: I know of a case of congenital hydrocephalus, which was tapped and then strapped. Strapping resulted in convulsions and had to be abandoned.. It is a question where—as in Dr. Ayres' case —the equilibrium between epithelial secretion and absorption is destroyed, whether any permanent result is attained by evacuation without the destruction of the epithelial lining of such cavities. I am on the negative side of the question. In my opinion the fluid will be reproduced and with this will come the symptoms present before the evacuation.

Dr. Lippincott: The restoration of sight so speedily after Dr. Ayres' operation is quite interesting. It was undoubtedly due to relief of pressure in the sub-dural space, Dr. Thomas' remarks remind me of the pathological analogy between Dr. Ayres' case and glaucoma. There we have the same sort of disturbance of the equilibrium between secretion and absorption. No amount and frequency of tapping has ever resulted in anything but temporary good. More than tapping must be done to accomplish a cure. It requires removal of a portion of the iris.

Dr. Lange: Exudations and transudations, inflammatory products and dropsical effusions are produced by congestions, active or passive; if epithelial activity was concerned in the production of the fluid in Dr. Ayres' case, as is assumed by Dr. Thomas, why need this factor alone be made responsible for the effusion? It is well established that large pleuritic effusions, not purulent, are frequently absorbed after removal of a sufficient quantity to abolish pressure. It is the pressure which constitutes the obstacle to absorption in such cases, and its removal restores to the absorbents their function. This restoration of a pleural cavity has been observed with sufficient frequency to warrant the belief that without the trocar, absorption would not have happened.

Dr. McCann: I am of opinion that Dr. Ayres' case was one of deep local abscess of the brain. There was very marked paralysis. A recurrence of the symptoms removed by the operation also points to this. The history of brain abscess is that symptoms repeat themselves with frequency, continuing for months and months, and resulting in imbecility. I would ask Dr. Ayres why he selected the point upon the skull which he did for perforation of the skull. It is not a point favorable to free drainage. Dr. Ayres' trephine, also, was very small. In removing tumors the bone section should be large. The benefit received by the doctor's patient was due, in my opinion, not to the fluid which was removed, but to the evacuation of an abscess. The drainage employed was certainly beneficial; the question is, should not a drainage tube have been inserted? The case is an additional argument in favor of brain surgery.

Dr. Stewart: There is a point in the etiology of internal hydrocephalus which has not been mentioned, namely, an obstruction to the communication of the ventricular cavities with the sub-arachnoid spaces of the brain and spinal cord, such as may occur at the aqueduct of Sylvius, which is naturally very narrow, or from closure of the foramen of Majendie. This, it seems to me, would explain some of the points of the case, namely, the gradual reaccumulation of the fluid and the small amount of fluid removed by the canula. A considerable amount of fluid exists in the sub-arachnoid spaces of the brain and spinal cord, and had there been a communication between the sub-arachnoid space and the ventricles, it is but natural to infer that there would have been a considerable amount of fluid withdrawn from this space instead of but little, as in this case, where only six drachms was removed. As regards the method of opening the ventricular cavity, Langenbeck has operated by thrusting a canula through the orbital plate of the frontal bone. This method, it seems to me, would obviate the objections of Dr. McCann regarding the manner of draining the cavity.

Dr. Ayres: In reply to Dr. McCann, I wish to say that I cannot call the case one of abscess. The fluid evacuated was clear and watery, and though we did not examine it under the microscope, we did make a chemical examination of it and found it contained no albumen. The specific gravity was not taken, as not enough fluid had been removed. But I never thought of an abscess, because it seemed perfectly clear to me that the case was one of pressure on the brain from accumulation of fluid. I made the small opening merely because I knew it would not require a large opening. We selected that point because it was the one most spoken of by authorities. As I said, I could find no regular cases of trephining. I thought it a safe point, and as the result proved, it was safe.

Dr. Hersman: It is considered unsafe to draw off more than two ounces of fluid. In Dr. Ayres' case we removed an ounce and the drainage evacuated considerable more. As to the point of operation, it was fixed by the experience of prior operators, and the result proves that no injury was done by it. The subsequent drainage evacuated probably six ounces, perhaps more. The quantity of fluid in the patient's head was certainly more than is normal. The restoration of the sight—absent before the operation — is conclusive evidence that this absence was caused by pressure, i. e., by a greater than normal amount of fluid in the head.

Dr. Hitzrot reported a case of

CYANOSIS AND DEATH OF INFANT.

Was called January 2d to a woman said to be in labor. She had 3 living children and had had two miscarriages. Her former labors had been normal. Her family history was excellent. No syphilis nor epilepsy on her nor on her husband's side. I found that she was not in labor, but was suffering with painful convulsive contractions of the muscular system of the whole body, with entire absence of uterine contractions. These pains recurred every 20 or 30 minutes. I considered them epileptic and gave her full doses of the bromides, which controlled them while she remained under the influence of the drug. On January 22d, upon which day she had a series of attacks, she was delivered, after a normal labor, of an eight-pound male infant. She had a few attacks during three following days, after which they recurred no more. She made a good recovery. The infant was of a natural color when born, but rapidly this became cyanotic, and then decidedly blue. He had a peculiar, plaintive whine which was constant. There was a slight bulbous enlargement of the finger ends. The infant lived seven hours, took a little sweetened water, but passed neither fæces nor urine. A post-mortem examination was not had. Whether the cyanosis was due to pulmonary stenosis, or to perviousness of the foramen ovale, is immaterial; the interest in the case centres upon the connection, if any, between the epileptiform convulsions of the mother and the anatomical cause of the cyanosis of the infant.

SIX MONTHS' FŒTUS BORN WITH UNRUPTURED MEMBRANES.

Dr. Batten reported a case, where, after prolonged moderate hæmorrhages, a six months' fœtus was born, encased in the membranes. These were at once ruptured, but the child was dead.

PASSIVE MOTION IN FRACTURES ABOUT JOINTS.

Dr. Lange: I desire to report two cases of fracture of the astragalus, first, because fracture of this bone is not a common one, but more particularly, as illustrating the good effects of immobilization. The first case happened in a young man, in consequence of a ten-foot perpendicular jump. This patient walked a distance of three blocks after the accident. He denied the existence of a fracture. He treated himself with liniment and lotions. His ankle-joint was not put at rest. The other patient, a man of 37, fell from an elevator, a distance of 30 feet, striking upon his right foot. His ankle joint was put into plaster under extension. The plaster was removed in four weeks, and three months after the accident his ankle was strong, useful, painless, mobile, and without visible deformity. The first case, now nearly two years old, presents considerable stiffness and enlargement of the joint, with pain on motion. It resulted in a painful and enlarged joint. The very great difference in the result of these two cases is to be ascribed to the difference of treatment, and illustrates the beneficial effect of immobility in this fracture.

Two years ago I dressed an arm for fracture of the internal epicondyle of the humerus, which happened a young lady by a fall from her horse. The dressing, although frequently removed, was worn for four weeks. After this time passive motion was employed, and the result was, and still is, that the arm cannot be entirely extended. There is deformity. The arm cannot be made quite straight, and there is thickening of the condyle. When I reported this case to the Society, the opinion was pronounced that immobilization of the joint had been too long continued, that if passive motion had been instituted earlier, deformity might have been avoided. This opinion was not unanimous, however; it was also held that four weeks' quiet of the joint was the best treatment for the fracture.

Finally, two cases of Colles' fracture have been for me very instructive, as examples of the result of early passive motion. In one of these cases I removed the splints on the tenth day, in the other on the fourteenth day. Passive motion was not, however, immediately employed. The wrists were simply released, and patients instructed to move the fingers. When the splints were removed the swelling had disappeared, and the temperature of the arms was no longer elevated. But swelling and temperature elevation returned because of removal of the splints so early, because of the lack of immobilization. Motion so early is a most powerful irritant. And irritation, evidenced by swelling and temperature elevation, is synonymous with additional deposits about, between and upon muscular sheaths and tendons which later become organized, and prohibit normal movement. Of all Colles' fractures I

have treated, these two, as far as usefulness of the hand is concerned, are the worst. This is not true of the characteristic deformity—the prominence of the ulna, which results more from the nature and complexity of the fracture than from its treatment, but it is true of the fibro-cellular ankylosis, which determines the amount of permanent damage to finger and wrist movement. I have no doubt the early removal of the splints alone is responsible for the result in these cases.

Dr. Batten: In my opinion, a patient with Colles' fracture will get along better without splints than with them. I have removed the splints in two weeks, and made passive motion. I have always removed the splints in three weeks. I have dressed this fracture simply with adhesive strips, and had a better result than with splints. Fingers, wrist, elbow and shoulder, all grow stiff with splints.

Dr. Kearns: Many years ago I treated a case of Colles' fracture with a straight splint with an elegant result. For many years now I treat all Colles' fractures, all fractures of the radius and of the fore-arm, with my flexed splint. My splint carries out the most important principle of the treatment of fractures at the wrist, namely, complete relaxation of all the muscles of the hand and fore-arm. My splint retroflexes the hand, relaxing all the muscles on the dorsum of the arm and flexes the fingers, relaxing all the flexors on the opposite aspect of the arm. This is the principle of the splint, perfect muscular relaxation as well as fixation of the bones.

Dr. Murdoch: The cases of fracture in the vicinity of joints, related by Dr. Lange, well illustrate the advantages of rest, and the immobilization of the fractured bones until union has taken place. They also show the evils which may result from a too early resort to passive motion. I fully agree with Dr. Lange in what he has said. This is a very interesting question, namely: When should passive motion be commenced after fracture near a joint? Prof. Hamilton and other eminent authors have said that in treating fractures in the vicinity of the elbow-joint, that passive motion should be commenced after a week or ten days from the receipt of the fracture; and he also says that in many cases these fractures could be best treated without any splint at all. It was my custom in the first years of my practice to follow this teaching. And in following it, and in seeing it practiced by others, I have seen joints becoming stiffer and stiffer until complete ankylosis of the joint has occurred. More extended experience has fully convinced me that the practice is altogether wrong. It is contrary to the first principles by which all injured parts should be treated. Rest of any part of the body which has met with a severe injury is the first principle of treatment. This is especially true of fractures. The fracture should be *first* reduced, and, *second*, should be retained in position. And no motion of any joint should be permitted which might displace the fragments. The immobilization should be maintained until union between the fragments has taken place, which can only occur after three or four weeks. Then passive motion of the very gentlest kind, and motion which does not cause any pain, may be permitted. A departure from this practice can only result in displacing the fragments, cause inflammation in the sheaths of the tendons, favor an effusion of lymph, a too abundant callus, and ankylosis of the joint.

Dr. McCann: Dr. Murdoch has voiced my opinion regarding rest and passive motion in fractures about joints. Stiffness from fractures about the elbow joint occurs not from want of passive motion, but because in young persons small bone fragments unite wherever they may happen to be placed by the violence of the fracture. Then it may happen that new bone, bone uniting the fragment to the shaft, will be formed and forever impair the usefulness of the joint, particularly the elbow joint.

This happens no matter what kind of splint may have been used, straight, flexed, or simply a sling. You will occasionally get a bad result in spite of all or any splint you may use, in spite of passive motion. I believe, however, that all fractures should be kept at rest until union has been accomplished. After this adhesions may be broken up by gentle passive motion. The only other thing I wish to say is that I hope the Society will not endorse the principle that Colles' fractures should ever be treated without a splint. It would under no circumstances be safe to state to a jury that no splint had been used in the treatment.

Dr. Thomas: I believe fractures should be kept at complete rest until united. Colles' fracture can be well and properly treated with almost any splint. The multiplicity of splints for this fracture proves it. The final result will depend upon the amount of injury, besides that done the radius, which the accident has inflicted. If the bone only is broken the result will be good. If, in addition, the ulna is dislocated, then it will be less good; and other additional injuries will have their weight in determining the final result.

Dr. Lange: I am sorry I failed to emphasize the point I wished to bring up for discussion. It was whether passive motion should be made after a week or two. Those gentleman who have spoken this evening have decided with me. I think it bad practice to make passive motion early in the treatment of fracture about joints.

Dr. Buchanan: It has occurred to me that the value of special splints and other apparatus used to maintain apposition of broken fragments of bone is often overrated. I am reminded of a case of fracture of the clavicle, in which the patient removed the dressings in a few days, despite the warnings of a possible deformity, and returned to his work. He had, however, good apposition, good union and no excess of callus. This and other similar cases have made me somewhat skeptical of the absolute necessity or even the advisability of many of the immovable dressings now in use. Rest of the bone is unquestionably the great desideratum, but the value of much of the cumbrous apparatus used might be questioned. In this connection I would remark, since the treatment of Colles' fracture without a splint, after the method of Dr. Moore, of Rochester, has been mentioned, that if this is a good method of treatment, it should be adopted irrespective of the legal responsibility involved.

Dr. Murdoch, in commenting upon the case related by Dr. Buchanan, said, that fractures will unite even when a good deal of motion is permitted between the fragments, and even when the fragments are somewhat asunder is well known. It is well known that the fractures which the lower animals receive in their combats with each other, and as the result of falls, readily unite, notwithstanding that they are not accurately adjusted, or maintained in position. So also in man, in bones which it is impossible to keep at rest (as the ribs) do they unite. But the union which is effected in these cases is by a very abundant supply of callus. In these cases there is always a great amount of provisional callus, a large ensheathing callus.

In the case of a fractured clavicle, when the fragments have not been accurately held in position, the only evils which result are the deformity and the greater length of time for union. The manner in which fractures unite, where the adjustment is accurate and the immobilization perfect, is entirely different. It is probable that in these rare cases union will take place as it does in the soft parts, namely, by first intention. In these cases there is no provisional callus. Where provisional callus exists motion between the fragments has been permitted, and the amount of callus will be in direct proportion to the amount of separation and motion.

From what has been said it is evident that, although accurate adjustment and motion between the fragments will not always prevent the union of bones, it will always delay the union, and result in some deformity of the bone. The resultant deformity may not be a matter of great moment in some bones and in some situations, but when it occurs in, or near a joint, it is likely to seriously interfere with the motion of the joint and may preduce bony ankylosis. It is for these reasons that in all cases (and especially in the treatment of fractures in the vicinity of joints), that the adjustment should be as perfect as possible and no motion permitted until there is some union between the fragments.

